# Dextran sulfate inhibits acute *Toxoplama gondii* infection in pigs

**DOI:** 10.1186/s13071-016-1421-9

**Published:** 2016-03-09

**Authors:** Kentaro Kato, Yuho Murata, Noriyuki Horiuchi, Atsuko Inomata, Mohamad Alaa Terkawi, Akiko Ishiwa, Yohsuke Ogawa, Shinya Fukumoto, Fumikazu Matsuhisa, Kenji Koyama

**Affiliations:** National Research Center for Protozoan Diseases, Obihiro University of Agriculture and Veterinary Medicine, Inada-cho, Obihiro, Hokkaido 080-8555 Japan; Department of Veterinary Microbiology, Graduate School of Agricultural and Life Sciences, The University of Tokyo, 1-1-1 Yayoi, Bunkyo-ku, Tokyo, 113-8657 Japan; Department of Basic Veterinary Medicine, Obihiro University of Agriculture and Veterinary Medicine, Inada-cho, Obihiro, Hokkaido 080-8555 Japan; National Institute of Animal Health, National Agriculture and Food Research Organization (NARO), 3-1-5 Kannondai, Tsukuba, Ibaraki 305-0856 Japan

**Keywords:** Dextran sulfate, Intermediate host, Pig, Public health, *Toxoplasma gondii*

## Abstract

**Background:**

*Toxoplasma gondii* is a highly prevalent protozoan that can infect all warm-blooded animals, including humans. Its definitive hosts are Felidae and its intermediate hosts include various other mammals and birds, including pigs. It is found in the meat of livestock which is a major source of human infection. Hence the control of toxoplasmosis in pigs is important for public health. We previously showed that dextran sulfate (DS), especially DS10 (dextran sulfate MW 10 kDa), is effective against *T. gondii* infection both in vitro and in mice. In this study, we asked whether DS affects *T. gondii* infection of pigs, one of the main animal sources of toxoplasmosis transmission to humans.

**Methods:**

Fourteen-day-old male pigs (*n* = 10) were infected with *T. gondii* and then immediately treated with different doses of DS10; clinical, pathological, and immunological analyses were performed 5 days post-infection.

**Results:**

DS10 had an inhibitory effect on toxoplasmosis in pigs. Intravenous injection of DS10 prevented the symptoms of toxoplasmosis and reduced the parasite burden and inflammation induced by *T. gondii* infection. High-dose DS10 (500 μg per head) caused reversible hepatocellular degeneration of the liver; middle-dose DS10 (50 μg per head) was effective against toxoplasmosis in pigs without causing this side effect.

**Conclusions:**

Our data suggest that middle-dose DS10 led to minimal clinical symptoms of *T. gondii* infection and caused little hepatocellular degeneration in our pig model, thereby demonstrating its potential as a new treatment for toxoplasmosis. These data should be very beneficial to those interested in the control of toxoplasmosis in pigs.

**Electronic supplementary material:**

The online version of this article (doi:10.1186/s13071-016-1421-9) contains supplementary material, which is available to authorized users.

## Background

*Toxoplasma gondii* is a highly prevalent protozoan that can infect all warm-blooded animals, including humans. Its definitive hosts are Felidae and its intermediate hosts include various other mammals and birds, including pigs. In these intermediate hosts, *T. gondii* has two asexual stages: the tachyzoite stage and the bradyzoite stage. Tachyzoites cause toxoplasmosis in fetuses and immunocompromised patients. Bradyzoites multiply within tissue cysts that are found in the meat of livestock, especially pork and mutton, and they are a major source of human infection [1]. Hence the control of toxoplasmosis in pigs is important for public health.

Pigs acquire *T. gondii* by ingesting oocysts from a contaminated environment or tissue cysts from infected animals [2]. To date, there have been relatively few studies of *T. gondii* in pigs and, as a result, there is little information regarding the pathology of pigs infected with *T. gondii*. One study examined piglets infected with a low virulent cyst-forming strain of *T. gondii* and analyzed the immunological response to the infection [3]. Another study evaluated the safety of vaccination and the persistence and distribution of the *T. gondii* stages within tissues following vaccination [4]. Mouse bioassays, histopathology, and PCR have also been used to detect *T. gondii* infection in tissues from experimentally infected pigs [5]. The pathogenicity in 7-week-old pigs to five different *T. gondii* strains of various host species origin was compared after intravenous inoculation of tachyzoites in another study [6]. Pigs infected with tachyzoites, tissue cysts, or oocysts showed dose-dependent clinical effects such as loss of appetite, fever, and poor general condition [7]. Another study examined whether vaccination with the RH strain could induce protective immunity to oral challenge with *T. gondii* oocysts [8]. These researchers also studied the distribution of tissue cysts in porcine tissues, by feeding the oocysts of four strains of *T. gondii* to pigs [9].

With regard to the development of anti-*Toxoplasma* drugs, previous studies have shown that the attachment of *Toxoplasma* to the host cell is mediated by interactions with sulfated glycosaminoglycans (GAGs) on the host cell, and that excess soluble GAGs inhibit this attachment to various cell lineages [10]. Monteiro showed that the ability of *T. gondii* to infect Chinese hamster ovary (CHO) cells deficient in sialic acids was reduced by 26.9 % compared with wild-type cells, indicating that sialic acid is critical for attachment and invasion of *T. gondii* [11]. Micronemal proteins (MICs) are released onto the parasite surface before host cell invasion and play important roles in host cell recognition, attachment, and penetration. Structural analysis of TgMIC1 revealed a novel cell-binding motif called the microneme adhesive repeat region (MARR), which provides a specialized structure for glycan discrimination [12]. Carbohydrate microarray analyses have shown that TgMIC13 and TgMIC1 share a preference for α2-3- over α2-6-linked sialyl-N-acetyllactosamine sequences [13]. P104, a PAN/apple domain-containing protein expressed at the apical end of the extracellular parasite, functions as a ligand in the attachment of *T. gondii* to chondroitin sulfate or other receptors on the host cell, facilitating invasion by the parasite [14]. In our previous study, we assessed the effects of several GAGs on toxoplasmosis and revealed that dextran sulfate MW 10 kDa (DS10) was the most effective in inhibiting the acute infection in vitro. Moreover, DS10 also had an inhibitory effect on *T. gondii* infection of mice [15].

In the present study, we examined the pathological condition of pigs infected with *T. gondii*. The effects of DS10 on *Toxoplasma* infection of pigs were assessed *via* host clinical, pathological, and immunological analyses.

## Methods

### Cells and parasites

Vero cells were cultured in Dulbecco’s Modified Eagle’s Medium (DMEM) supplemented with 5 % fetal calf serum (FCS), L-glutamine, penicillin, streptomycin and 0.15 % NaHCO_3_. Tachyzoites of the wild-type *T.gondii* RH strain were maintained in monolayers of Vero cells in DMEM supplemented with 1 % FCS, L-glutamine, penicillin, streptomycin and 0.15 % NaHCO_3_ [15].

### Animal experiments

#### Ethical approval

All animal work has been conducted according to the national guidelines of Japan. The protocol was approved by the Committee on the Ethics of Animal Experiments of Obihiro University of Agriculture and Veterinary (Permit Number: 25–26).

**Fig. 1:**
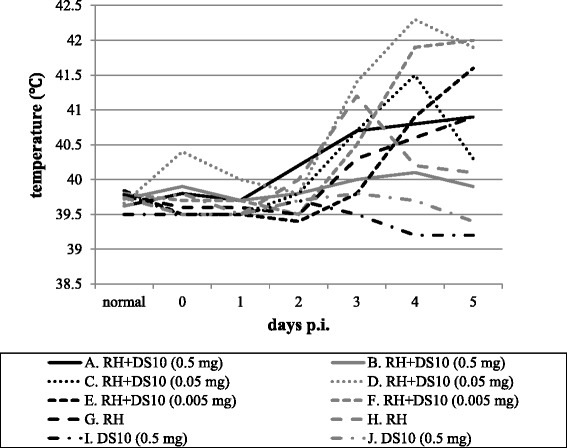
Body temperatures of the experimental pigs. Changes in body temperatures (°C) of experimental pigs were calculated and the days post-infection are shown. Pigs A and B were injected with *T. gondii* RH strain and 0.5 mg of dextran sulfate. Pigs C and D were injected with *T. gondii* RH strain and 0.05 mg of dextran sulfate. Pigs E and F were injected with *T. gondii* RH strain and 0.005 mg of dextran sulfate. Pigs G and H were injected with only *T. gondii* RH strain. Pigs I and J were injected with only 0.5 mg of dextran sulfate

Pig infection experiments were performed at the Research Institute for Animal Science in Biochemistry & Toxicology. Fourteen-day-old male pigs (*n* = 10) were used in this study. They were kept under specific pathogen-free conditions before purchase. Tachyzoites of the wild-type *T. gondii* RH strain were collected from infected Vero cells by using the syringe-lysis method and then filtered through a 5-μm pore filter [15]. Parasites were counted with a hemocytometer. Pigs were divided into the following five groups: the DS-H group (A and B), DS-M group (C and D), DS-L group (E and F), TG-control group (G and H), and DS-control group (I and J) (Fig. 1). The pigs of the DS-H group, DS-M group, and DS-L group were injected intravenously with 1 × 10^6^ tachyzoites in 1 ml of DMEM. At the same time, Dextran Sulfate MW 10 kDa (DS10) was administered intravenously to the pigs of the DS-H, DS-M, and DS-L groups; the DS-H group was injected with of DS10 500 μg per head, the DS-L group received 50 μg per head, and the DS-L group received 5 μg per head. The pigs of the TG-control group were injected with 1 × 10^6^ tachyzoites only, and the pigs of the DS-control group were injected with 0.5 g of DS10 and DMEM but no tachyzoites. We monitored the temperature and body weight of the pigs and collected sera from them until 5 days post-infection. Then, the pigs were euthanized and submitted for necropsy. Samples of liver, spleen, kidney, heart, lung, hilar lymph node, colon and brain were collected for qRT-PCR and pathological analyses.

### Antibody response

*Toxoplasma* antibody titers were determined using Toxo Check-MT according to the manufacturer’s instructions (Eiken, Tokyo, Japan).

### Serum biochemistry

Serum samples for biochemical analysis were examined with a clinical chemistry automated analyzer (Toshiba Medical Systems Co., Tochigi, Japan). Concentrations of total protein, sodium, potassium, chloride, albumin, glucose, cholesterols, aspartate transaminase, γ-glutamyl transpeptidase, creatinine and blood urea nitrogen in the serum samples were measured by using specific detection reagents (Denka Seiken, Tokyo, Japan).

### Quantitative real-time polymerase chain reaction (qRT–PCR)

Organs including spleen, liver, kidney, lungs, lymph nodes, heart and brain were harvested, placed in Trizol Reagent (Invitrogen, Carlsbad, CA, USA) and lysed for RNA and genomic DNA extraction in accordance with the manufacturer’s instructions. Total RNA was isolated by using the ReliaPrep Miniprep System (Promega, Madison, WI, USA) and then reverse transcribed to first-strand cDNA (Invitrogen, Carlsbad, CA, USA) following the manufacturer’s instructions. Parasite burden was determined in genomic DNA samples (adjusted to 50 ng/μl) by using an ABI Prism Genetic Analyzer (Applied Biosystems, Carlsbad, CA, USA) with SYBR Green (Applied Biosystems) and specific primers [15]. The expression of cytokines was analyzed by means of qRT-PCR with the specific primers listed in the (Additional file 1: Table S1). The primers for qRT-PCR were designed with the Primer Express software (Applied Biosystems). Specific gene expression was normalized to the expression of ubiquitin by using swine β-actin as the housekeeping gene. Relative gene expression was calculated by using the 2^−ΔΔCt^ method.

### Pathological analyses of experimental pigs

Collected samples were fixed in 15 % neutral buffered formalin, embedded in paraffin, sectioned at 5 μm, and stained with hematoxylin and eosin (HE) for histological examination. For objective observation, major lesions were counted and the number of lesion per unit area was calculated. The counted number was divided by the tissue area of the organ on a glass slide, which was measured by using image analysis software (BZX_Analyzer, version 1.1.0.1, Keyence Corporation, Osaka, Japan) to obtain the number of foci per unit area. The sections of liver and lung were also subjected to immunohistochemical examination. These examinations were performed using a rabbit polyclonal anti-*T gondii* antibody (1:50 dilution, Abcam, CA, USA, ab15170) as the primary antibody and the simple stain MAX-PO polymer reagent (Nichirei Bioscience, Tokyo, Japan) after autoclave pretreatment for antigen retrieval (121 °C for 20 min in 0.01 M citrate buffer, pH 6.0). For objective analyses, the number of positive reactions per unit area was calculated. Positive reactions were identified and counted according to the following criteria by two pathologists, who were not part of the live pig experiments or the necropsies and were blinded to the group name and pig ID: independent brown round to oval substance was counted as 1, regardless of its size; macrophages containing fine positive granules were counted as 1, regardless of the amount contained. The number of organisms was then divided by the tissue area of the organ on the glass slide.

### Pathological analyses of experimental mice

The tail veins of four mice were injected with 2.5 mg, 250 μg, 25 μg, or 2.5 μg of DS10 or with PBS, respectively, to analyze the toxic effects of intravenous injection of DS10 to animals. Thus, we examined a total 20 mice before performing the pig experiments with DS10. The body weights of the mice were calculated every day until autopsy at 5 days post-injection. The collected samples were subjected to HE staining for histologic examination.

## Results

### DS10 suppresses the symptoms of toxoplasmosis and reduces parasite burden in organs

To analyze the toxic effects of intravenous DS injection to animals, we performed pathological experiments with 20 mice before proceeding with DS experiments using pigs. We found that mice intravenously administered DS experienced neither significant weight loss nor pathological effects (data not shown). We, therefore, proceeded with the pig experiments.

**Fig. 2:**
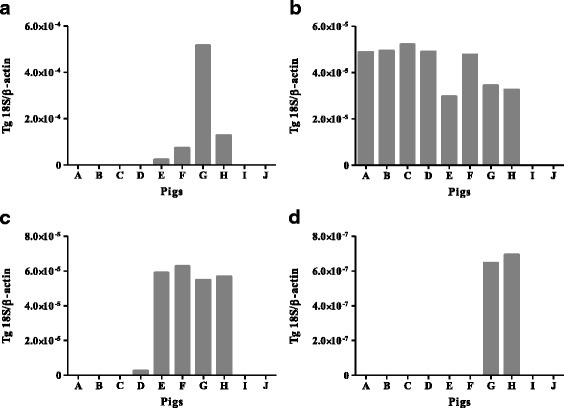
Parasite burden in the organs of individual pigs. Analyses of the relative expression of *T. gondii* 18S rRNA (by use of qRT–PCR) in liver (**a**), lung (**b**), lymph nodes (**c**) and kidney (**d**). Results represent the expression of the 2^ddCt^ parasite gene in pigs

On the day of pig inoculation, the average body weight of the ten experimental pigs was 4,199 g. All pigs infected with *T. gondii* tachyzoites (pigs A, B, C, D, E, F, G and H) showed symptoms 3–5 days after inoculation. In contrast, no clinical signs were seen with the non-infected pigs that were injected with DS10. The clinical signs exhibited by the pigs that suffered from acute infection with *T. gondii* were the characteristic signs of fever, loss of appetite, sneezing, and unkempt fur. These clinical signs were scored in the test pigs relative to the symptoms of the control pigs (I and J). Of note, the pigs that received the lowest dose of DS10 (5 μg per head) showed similar signs to those exhibited by the RH-infected control pigs (G and H), including fever (Fig. 1), loss of appetite, shivering, unkempt fur, and tachypnea. The clinical signs were less severe in the pigs that received the higher doses of drug, and the one pig that received the highest dose of drug (500 μg per head) showed no symptoms. These results suggest that high-dose DS10 suppressed the symptoms of toxoplasmosis. We then quantified the parasite burden in various pig organs by using qRT-PCR. Strikingly, all of the infected pigs exhibited high parasite burden in the lung samples but no parasites were found in the spleen or brain samples (Fig. 2). Parasites were detected in the liver and lymph node samples of pigs E, F, G and H and in the kidney samples of pigs G and H. These results indicate that treatment with DS10 reduces *T. gondii* burden in certain pig organs.

Next, we performed biochemical analyses of serum samples collected from the pigs on day 5 post-infection to assess the effects of treatment on organ function. Serum levels of GOT, GPT, γGTP and TG (as indictors of liver function) were within the normal ranges of healthy pigs for all of the pigs tested. In contrast, the serum CRE and BUN levels (as indicators of kidney function) were slightly elevated in pigs G and H (data not shown). These pigs exhibited symptoms of toxoplasmosis consistent with the high parasite burden in their livers and kidneys.

### Infection, but not treatment with DS, triggers inflammatory responses in organs

**Fig. 3:**
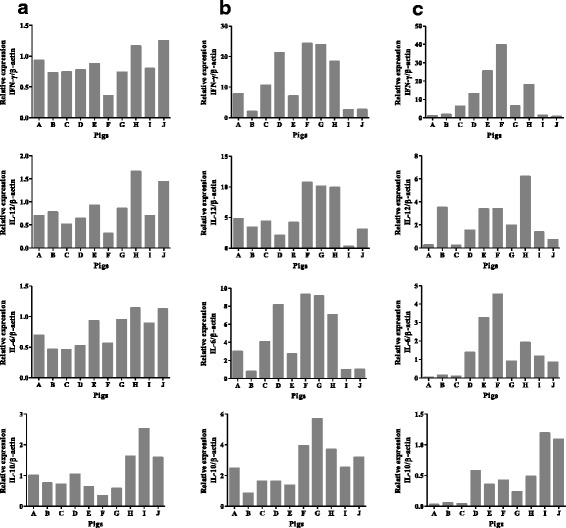
Cytokines gene expression in tissues of pigs. Analyses of the relative expression of IFN-γ, IL-12, IL-6 and IL-10 (by use of qRT–PCR) in liver (**a**), lung (**b**), and lymph nodes (**c**). Results represent the expression of the 2^ddCt^ parasite gene in pigs

Immunological responses were evaluated on day 5 post-infection in serum for antibody responses, and in liver, lungs, spleen, and lymph nodes for cytokine gene expression by using the latex agglutination test and qRT-PCR, respectively. Notably, the antibody responses were below the detection limits of the latex kit for all of the pig sera tested (data not shown). Gene expressions for IFN-γ, IL-12 and IL-6 were varied in the tested organs of the pigs; however, these levels were relatively high in lymph nodes and lungs as compared to those in livers (Fig. 3). These increases seemed to be consistent with the elevated parasite burden in lymph nodes and lungs. Control DS treated pigs exhibited relatively lower expression of IFN-γ, IL-12 and IL-6 cytokines in the lung and lymph nodes compared with pigs infected with *T. gondii* (Fig. 3, panels A-H). In contrast, IL-10 expression in liver and lymph nodes was high in control DS treated pigs (Fig. 3, panels I and J), and in lungs of pigs that received a lower dose of DS and no treatment (Fig. 3, panels F-H). These data may suggest that treatment with DS triggers only minor inflammatory responses in the tested organs.

### Treatment with DS alleviates hepatic and pulmonary lesions caused by *T. gondii* infection

To gain further insights into the effects of DS on the pathogenesis of *T. gondii* infection, we performed histological analyses of the pigs’ spleen, liver, kidney, lungs, lymph nodes, heart and brain. These histological analyses revealed hepatic and pulmonary lesions, and follicular reactions in the hilar lymph nodes.

**Fig. 4:**
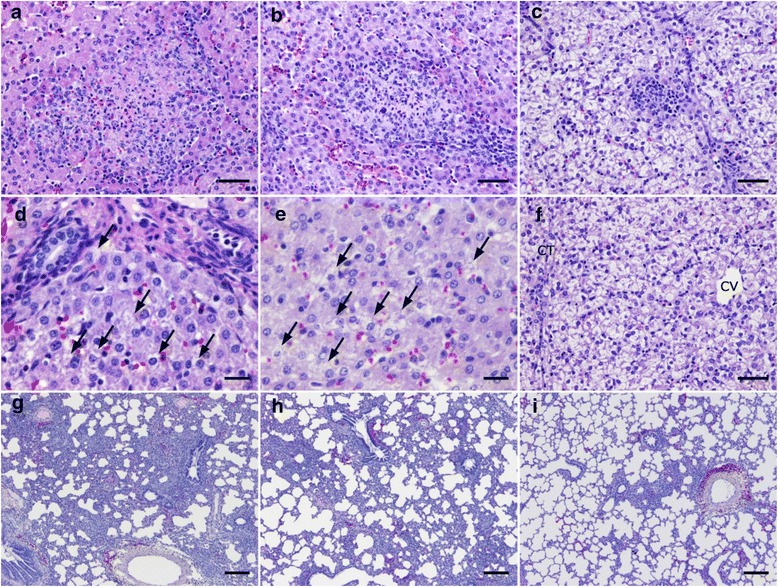
Histological features of the experimental pigs. Inflammatory and necrotic foci of liver (**a–c**), degenerative changes in hepatocytes (**d–f**) and pulmonary inflammatory foci (**g–i**) were stained with hematoxylin and eosin. **a** Liver, pig E in the DS-L group. Focal necrosis of hepatocytes with infiltrated inflammatory cells is evident. **b** Liver, pig D in the DS-M group. Similar necrotic and inflammatory foci as those shown in Fig. 4a can be seen. Note that these foci are smaller than those of Fig. 4a. **c** Liver, pig A in the DS-H group. Focally aggregated inflammatory cells without hepatocellular necrosis are shown. Hepatocellular degeneration is also evident. **d** Liver, pig F in the DS-L group. Mild vacuolar degeneration in a few hepatocytes (arrows) at the periphery of the hepatic lobe are shown. **e** Liver, pig D in the DS-M group. The stronger degenerative changes (arrows) were more frequently observed than those in Fig. 4d. **f** Liver, pig A in the DS-H group. Severe hepatocellular hydropic degeneration can be seen throughout the hepatic lobe. CT denotes interlobular connective tissue, and CV denotes the central vein. **g** Lung, pig E in the DS-L group. Extensive, moderately thickened alveolar wall due to infiltrated inflammatory cells was observed. **h** Lung, pig C in the DS-M group. The area of thickened wall is smaller than that in Fig. 4g. **i** Lung, pig A in the DS-H group. Diffuse, apparently normal alveoli and limited wall thickening were observed. Bars in **a**)–**c**) and **f**) represent 50 μm, bars in **d**) and **e**) represent 20 μm, and bars in **g**) and **h**) represent 200 μm

**Fig. 5:**
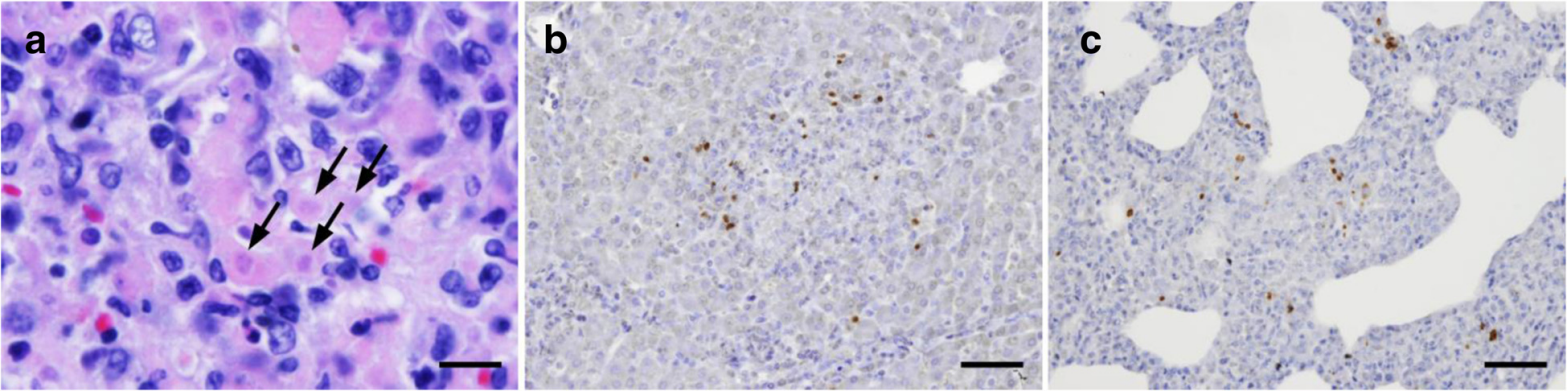
*T. gondii* organisms and immunohistochemical staining with an anti-*T. gondii* antibody. **a** Liver, pig E in the DS-L group. Slightly basophilic oval organisms (arrows) in the cytoplasm of hepatocytes were stained with hematoxylin and eosin. **b** Necrotic liver foci, pig E in the DS-L group. Positive reactions, stained brown, are scattered within the necrotic foci; non-affected parenchyma can be seen around the foci, as detected by immunohistochemical staining. **c** Lung, pig E in the DS-L group. Positive reactions were scattered throughout the thickened alveolar wall, as detected by immunohistochemical staining. Bar in **a**) represents 20 μm and bars in **b**) and **c**) represent 50 μm

Of note, multiple necrotic foci of hepatocytes with aggregated inflammatory cells were observed in the TG control and DS-L groups (Fig. 4a). The necrotic foci became narrower in the DS-M group (Fig. 4b). In the DS-H group, necrotic foci were not observed but a few small nests of aggregated mononuclear cells were scattered throughout the tissue (Fig. 4c). The inflammatory foci were observed in all pigs and were clearly more prevalent in *T. gondii-*infected pigs. *T. gondii* parasites were often observed within the necrotic foci in the TG-control, DS-L, and DS-M groups, but they were obscure in the HE specimen (Fig. 5a). No necrotic or inflammatory foci were observed in the DS-control group, whereas necrotic and/or inflammatory foci and reversible hepatocellular degeneration were observed in DS10-treated pigs. In the DS-L group, a few mildly vacuolated hepatocytes were observed at the peripheral zone of the lobule (Fig. 4d). In the DS-M group, moderate vacuolar degeneration of hepatocytes at the peripheral zone of the lobule was observed (Fig. 4e). In the DS-control and DS-H groups, severe hydropic degeneration was observed in almost all of the hepatocytes (Fig. 4f). No degenerative changes were observed in the TG-control group. Objective analyses of the hepatic lesions indicated that the necrotic foci were most abundant in the DS-L group, and that their numbers decreased as the dosage of DS10 increased.

In the lung, alveolar wall thickening due to mononuclear cell infiltration was observed. In the TG-control and DS-L groups, the lesions were moderate and diffuse (Fig. 4g). Moderate to mild or diffuse lesions were focally observed in the DS-M group (Fig. 4h), whereas the lesions were slighter and smaller in the DS-H group (Fig. 4i). Slight bronchitis and bronchocentric infiltration of mononuclear cells into the parenchyma were observed in all of the pigs including the DS-control group.

**Fig. 6:**
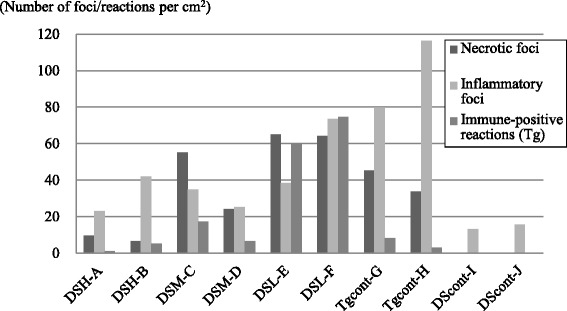
Necrotic, inflammatory foci, and positive reactions per unit area. The number of necrotic foci per square centimeter in the liver of each experimental pig was calculated and is shown as the left-hand bar (A and B, DS-H group; C and D, DS-M group; E and F, DS-L group; G and F, TG-control group; I and J, DS-control group). The number of inflammatory foci per square centimeter in the liver of each experimental pig was also calculated and is shown as the middle bar. The average number of immuno-positive reactions against the anti-*T. gondii* antibody per square centimeter in the liver of each experimental pig was calculated and is shown as the right-hand bar. Data represent the observations of two independent investigators

Immunohistochemical analyses using the anti-*T. gondii* antibody revealed that the suspected *T. gondii-*like organisms reacted positively. In addition, small granules within hepatocytes and infiltrated macrophages were found to give positive reactions. In the liver, the positive reactions were mainly found within necrotic foci, but were sometimes found within non-affected parenchyma (Fig. 5b). In the lung, the positive reactions were found within both the thickened alveolar wall and in non-affected pulmonary parenchyma (Fig. 5c). No positive reactions were detected in the DS-control group. The number of positive reactions appeared to decrease as the number of inflammatory or necrotic foci decreased in the liver. The calculated positive reactions per unit area in the liver decreased as the dosage of administered DS10 increased, according to the histological observations (Fig. 6). In addition to the number of necrotic foci, the number of positive reactions in the DS-L group was greater than that of the TG-control group. The calculated positive reactions per unit area in the lung also decreased as the dosage of administered DS10 increased, according to the histological observations (data not shown).

## Discussion

Infected pigs and pork products are important sources of *T. gondii* infection for humans and other animals. The discovery and development of effective drugs to treat these infections is therefore particularly important for the pork industry [2]. In the present study, we evaluated the effects of intravenous administration of DS10 to pigs experimentally infected with *T. gondii*. Treatment with various doses of DS10 was effective in reducing the clinical symptoms and parasite burden in the organs of the infected pigs. These results are consistent with our previous data showing that DS10 inhibits the growth of parasites in vitro and in vivo in a mouse model [15]. One possible explanation for our findings is that DS10 restricted the growth of the parasite in the lungs and thus prevented its spread to other organs. Indeed, previous studies have shown that lung is the first organ that is preferably invaded by *T. gondii* in experimental infections [16, 17]. Moreover, a related study showed that parasite burden is highest in the brain tissue followed by lungs and muscles in pigs after experimental infection [17]. The absence of parasite burden in the brain tissue in our study probably reflects the date of sampling (5 days post-infection). When we sampled later in the previous experiment, we detected parasites in this tissue.

In this study, we administered DS intravenously, which is not the route of drug administration that is widely utilized in animal husbandry. So, we need additional experiments to determine how effective oral administration would be against infection. Likewise, we only tested the effect of DS on the acute infection. Experiments on chronic infection would be needed when we foresee the real world usage of this treatment.

Our analysis of mRNA cytokine profiles revealed that the up-regulation in gene expression of pro-inflammatory cytokines was consistent with the elevated parasite burden in the lymph nodes and lungs of the pigs. Moreover, low IL-10 gene expression was detected in all studied pigs. In general, experimental infections with *T. gondii* elicit a dominant Th1 response, characterized by high production of IFN-γ and IL-12 during the acute stage of the infection, and Th2-associated cytokines, such as IL-4 and IL-10, which appear relatively late in infection to limit immune pathology [18–20].

In the current study, histopathological lesions were observed in the liver and lungs of all of the *T. gondii*-infected pigs; multi-focal necrotic foci and inflammatory foci in the liver as well as interstitial pneumonia were observed. These lesions were similar to those previously reported in naturally infected pigs [2, 21, 22]. Generally, experimental *T. gondii* infection rarely causes histopathological lesions in pigs [3, 5, 7, 9, 23], and only one report has described necrotic or inflammatory lesions of visceral organs [6]. *T. gondii* parasites were immunohistochemically detected in the liver and lungs, mainly within the lesions. Taken together, our data suggest that infection of juvenile piglets *via* intravenous administration of *T. gondii* provides a relevant model for investigating acute phase infection, producing similar pathological lesions to those observed in natural infections. The severity of the hepatic and pulmonary lesions and the amount of detected *T. gondii* antigen were remarkably different among animals and dependent on the injected dosage of DS10. Severe lesions and high parasite burdens were observed in pigs that received low-dose DS10, but the number of lesions and the burden gradually decreased as the dosage of the drug was increased. These results demonstrate that it is important to dose DS10 appropriately to obtain its inhibitory effect on *T. gondii* growth in pigs. Moreover, DS10 exhibited its degenerative effects on pig hepatocytes in a dose-dependent manner, suggesting that high-dose DS10 may require strict attention to limit its side effects. No inflammatory changes were detected in the colon despite previous reports that oral administration of dextran induces colitis in pigs [24]. Of note, Kim et al. [25] previously reported that intravenous injection of DS10 does not trigger any signs of colitis. Any side effect of DS10 is likely dependent on the dosage as well as the injection route.

## Conclusion

Our study revealed that DS10 is a promising agent for the treatment of toxoplasmosis, although high doses can cause adverse effects on hepatic cells in pigs. Our data suggest that a dose of approximately 0.05 mg of DS10 per average body weight of 4,199 g is an effective dose for swine because it led to minimal clinical symptoms of *T. gondii* infection and caused little hepatocellular degeneration in our pig model. In this study it is possible to potentially reduce parasite burden and cytokine up-regulation but further work is necessary to identify the drug targets or future inhibitors. These data should be very beneficial to those interested in the control of toxoplasmosis in pigs.

## Additional file

Additional file 1: Table S1. Primers used in the study. (DOCX 14 kb)
