# Supplementary material for: Dextran sulfate inhibits acute Toxoplama gondii infection in pigs
Source: Parasit Vectors. 2016 Mar 9;9:134. doi: 10.1186/s13071-016-1421-9 (PMC4784389; doi:10.1186/s13071-016-1421-9)
Supplement: Additional file 1: Table S1. — Primers used in the study. (DOCX 14 kb) [file 13071_2016_1421_MOESM1_ESM.docx]

Supplementary information

**Table S1. Primers used in the study.**

| **Target gene** | **Forward (5' – 3')** | **Reverse (5' – 3')** |
| --- | --- | --- |
| *INF-γ* | CGTGACTTTGTGTTTTTCTGGC | TCTTTAAAAAAGGGCGCCTG |
| *IL-12* | GCACAGTGGAGGCCTGCTTA | GGCAACTCTCATTCGTGGCT |
| *IL-6* | AGTCCAGTCGCCTTCTCCCT | GAAGGCAGTAGCCATCACCAG |
| *IL-10* | TGCCAAGCCTTGTCAGAGATG | CGGCATTACGTCTTCCAGGT |
| *β-actin* | CTCCATCATGAAGTGCGACG | GTGTTGGCGTAGAGGTCCTTG |
